# Wealth accumulation in rotation forestry – Failure of the net present value optimization?

**DOI:** 10.1371/journal.pone.0222918

**Published:** 2019-10-18

**Authors:** Petri P. Kärenlampi

**Affiliations:** Lehtoi Research, Lehtoi, Finland; College of Agricultural Sciences, UNITED STATES

## Abstract

The rate of wealth accumulation is discussed, and an expression for a momentary rate of capital return is presented. An expected value of the wealth accumulation rate is produced. The return rates depend on any yield function. Three different yield functions are applied, two of them published in the literature, and a third one parametrized using a comprehensive growth model. A common economic objective function, as well as a third known objective function, are applied and compared with the clarified wealth accumulation rate. While direct optimization of wealth appreciation rate always yields best results, procedures gained by maximizing the internal rate of return are only slightly inferior. With external discounting interest rate, the maximization of net present value yields arbitrary results, the financial consequences being at worst devastating.

## Introduction

In forest economics, optimization of forest management most commonly happens through maximization of net present value of future revenues [1, 2, 3, 4]. A discounting interest rate is applied in order to compute the present value of future incomes and expenses [5, 6, 7, 8, 9, 10, 11]. The discounting interest may vary over time [12, 13, 14, 15]. It has been stated that uncertainty induces declining discount rates along with time [16, 12, 17, 18]; however, it can be shown that there is an opposite effect on prolongation interest. Risk of destructive events has been considered as a premium to discount interest [19, 20]. Evolution of prices, as well as fluctuations in growth and prices, may be added [13, 21]. Taxation does contribute, as well as personal financies [22, 23, 24]. An imperfect capital market has been addressed [25]. A Hamiltonian formulation is available [26, 27]. Regardless of other details, the financial soundness of any operation depends on the choice of the discounting interest rate.

Risk of destructive events in forestry apparently has lately been exaggerated by changing climate, obviously enhancing the risk of wildfires, storm disasters, and biological damages [28, 29, 30]. Such increased risk factors can be accounted for by increasing discounting interest rate, even if the interest increase might be of arbitrary magnitude. A problematical factor is that increased discounting interest rate tends to reduce capitalization, and consequently reduce the rate of carbon sequestration [31, 32].

We are aware of one method in the determination of profitability in multiannual growth without an external discount rate [33, 34]. Sound management is supposed to maximize internal discounting interest (or internal rate of return), harvesting income being discounted to cover initial investments [33, 34]. There are a few problems related to this approach: it cannot be applied in the case of zero initial investment, and it does not consider the shape of the yield curve in any way. The latter deficiency is related to the fact that being established on cash-flow basis, the internal interest discounting cannot account for any time-variable capitalization effect.

In this paper, a method for determination of wealth accumulation rate in forestry is established. First, a momentary capital return rate function is formulated. Then, an expected value of the capital return rate is clarified. The return rates depend on any yield function. Three different yield functions are applied, two of them previously published in the literature [10], the third one created by specifying boundary conditions for a comprehensive growth model [35, 36].

The idea of clarifying capital return rates in forestry is not new [37]. However, the idea of clarifying expected values of capital return rate on the basis of a yield function appears to be a recent [38]. The consequences of maximizing discounted net present value of future revenues [1, 2, 3, 4] on the wealth accumulation rate have not been previously investigated.

We first present a more general formulation for the wealth appreciation rate, but later focus in stationary rotation forestry. Stationary forestry here means that the distribution of stand ages does not evolve. This is practically possible if the stand ages are evenly distributed, and the same duration of rotation cycle is applied to stands. A concept “normal forest” has been previously applied to such a structure [39]. Stationary forestry here also implies that prices and expenses do not evolve. We are naturally discussing prices and expenses in real terms.

Rotation forestry here refers to a system with periodic temporal boundary conditions. In other words, at the end of any rotation cycle, the system returns to a state which equals the initial state. Then, a new rotation cycle starts. As an idealization of reality, the cycles are assumed to be similar to each other. In order to promote simplicity, we do not discuss any intermediate harvesting entries during a rotation cycle.

The structure of the remaining part of the paper is as follows. First, a basic notation of wealth accumulation is introduced, along with an application to stationary rotation forestry. Then, the three different economic criteria (objective functions) for the optimization of forest management are introduced. Thirdly, the three different yield functions are specified. Two of the yield functions are even-age forestry models, the third is an uneven-age forestry model. Then, the outcome of the three different objective functions is reported, applied to any of the three yield functions. Finally, the three different forest economics approaches are compared from the viewpoint of wealth accumulation.

## Methods

### Wealth accumulation

Let us first introduce a very basic notation of wealth accumulation:

$$W\left(T_{2}\right)=W\left(T_{1}\right)+\int_{T_{1}}^{T_{2}}\frac{dW}{dt}dt=W\left(T_{1}\right)+\int_{T_{1}}^{T_{2}}W\left(t\right)r(t)dt,$$ (1)

where *W*(*T*_1_) is wealth at time *T*_1_, *W*(*T*_2_) is wealth at time *T*_2_, and *r* is a relative wealth increment rate. Provided the time is expressed in years, *r* becomes the relative annual wealth increment rate. In the absence of withdravals, Eq (1) can be rewritten

$$W\left(T_{2}\right)=W\left(T_{1}\right)\exp\left[\int_{T_{1}}^{T_{2}}r(t)dt\right].$$ (2)

The above equations describe the total amount of wealth. It possibly should be constituted from stand-level capitalizations, given per unit area. By definition, the expected value capitalization per unit area is

$$\langle \kappa \rangle =\int_{0}^{\infty }p(\kappa )\kappa d\kappa ,$$ (3)

where *p*(*κ*) is the probability density function of capitalization *κ*. By change of variables we get

$$\langle \kappa \rangle =\int_{0}^{\tau }p(\kappa )\kappa \frac{d\kappa }{da}da=\int_{0}^{\tau }p(a)\kappa (a)da,$$ (4)

where *a* is stand age, and τ is rotation age. The expected value of the increment rate of capitalization is

$$\langle \frac{d\kappa }{dt}\rangle =\int_{0}^{\tau }p(a)\frac{d\kappa (a)}{dt}da.$$ (5)

Correspondingly, the expected momentary rate of relative capital return is

$$\langle r(t)\rangle =\frac{\langle \frac{d\kappa }{dt}\rangle }{\langle \kappa \rangle }=\frac{\int_{0}^{\tau }p(a)\frac{d\kappa (a,t)}{dt}da}{\int_{0}^{\tau }p(a)\kappa (a,t)da}=\frac{\int_{0}^{\tau }p(a)\kappa (a,t)r(a,t)da}{\int_{0}^{\tau }p(a)\kappa (a,t)da},$$ (6)

which can be readily substituted to Eq (2). Eq (6) can be applied to whatever estate provided the functions appearing in it can be reasonably approximated.

It is worth noting that in Eq (6), there is a notation of stand age (*a*) and a notation of time (*t*). The probability density of stand age *p*(*a*) could vary as a function of time. However, constancy of the stand age distribution is a real possibility if there is an even distribution of stand ages, varying between zero and the rotation age τ.

It is indicated in Eq (6) that the capitalization *κ*(*a*,*t*), as well as the momentary capital return rate, *r*(*a*,*t*) are functions of time, in addition to stand age. This would be the case if there would be an appreciation of real estate values, for example, and an eventual appreciation of real estate values might considerably contribute to financial sustainability [40]. However, in the remaining part of this paper, we discuss forestry in terms of stationary capitalization per unit area. In addition to the assumption of the constancy of the stand age density function, this corresponds to assuming that wood stumpage prices, as well as real estate prices, develop along with general development of prices and expenses. Then, the capital return rate *r*(*a*) corresponds to real return, excluding eventual inflation of prices.

Now, in the case of constant capitalization per unit area, Eq (2) becomes

$${\left(\frac{W\left(T_{2}\right)}{W\left(T_{1}\right)}\right)}^{\frac{1}{T_{2}-T_{1}}}=e^{\langle r\rangle }.$$ (7)

In other words, the expected value of the relative wealth increment rate 〈*r*〉 is a unique measure of wealth accumulation, regardless of the time horizon, and it is given by

$$\langle r\rangle =\frac{\int_{0}^{\tau }\frac{d\kappa (a)}{dt}da}{\int_{0}^{\tau }\kappa (a)da}.$$ (8)

Eq (8) is a simplified form of Eq (6), created assuming that the probability density function of stand ages is a constant function. It is worth noting that even apart from the “normal forest” boundary condition [39] the constancy of the probability density of stand age within the integration range is valid when discussing one single stand.

### Objective functions to be compared

Probably the most commonly used objective function used in forestry optimizations is maximization of net present value of future revenues and expenses [1, 2, 3, 5, 6, 7, 8, 9, 10, 11]:

$$NPV_{a=0}=\int_{0}^{\tau }R(a)e^{-ia}da\frac{1}{1-e^{-i\tau }},$$ (9)

where *R(a)* corresponds to net proceeds at age *a*, *i* is discounting interest rate, and τ again is rotation age. The last factor after the integral corresponds to discounting of further growth cycles, each of rotation age τ. Management decisions based on Eq (9) can be readily compared with those based on Eq (8), under the boundary condition of stationary capitalization per unit area.

A significant problem in Eq (9) is that it contains the external discount rate *i*. The discount rate is external in the sense that it is unrelated to the capital return within the growth process, and consequently subject to arbitrary changes. Another issue in Eq (9) is that it does not consider the shape of the yield curve in any way. In other words, Eq (9) discusses revenue on cash basis, instead of financial grounds.

The problem of arbitrary external interest has been resolved by maximizing an internal rate of return. As introduced by Newman [34, 33], it is determined for a period of duration according to the criterion

$$\int_{0}^{\tau }R(a)e^{-oa}da=0.$$ (10)

The general form of Eq (10) does not appear to make sense at first glance. However, the Equation makes complete sense provided negative net proceeds appear at the beginning of the rotation, and positive net proceeds are generated by the end of the growth cycle. It is sometimes stated that maximizing the internal rate of return would not consider any bare land value. Such a statement, however, is incorrect. Eq (10) considers one single rotation, whereas Eq (9) refers to maximization of long-term discounted proceeds. Optimization of one single rotation possibly should consider purchase of bare land at the beginning, and sales of it at the end. Even if actual real estate transaction would not be implemented, the purchase price should be considered as an opportunity cost of not selling the bare land in the beginning. By the end of the single rotation to be optimized, the bare land constitutes a saleable asset.

It is worth noting that the only Equation above that requires periodic boundary conditions in time is Eq (9).

Evidently, a third objective function to be compared from the viewpoint of wealth accumulation should be the maximization of the expected value of the relative wealth increment rate 〈*r*〉, given in Eq (8). Such an objective function has been recently derived in terms of a state-space approach [38]. It might be considered trivial that this criterion is superior from the viewpoint of wealth accumulation, considering that it has been derived as the unique measure of wealth accumulation rate at Eqs (1…7). However, recent discussion has indicated that this is considered far from trivial. Regardless of the eventual triviality, it is of interest how much inferior objective functions (9) and (10) might be, in comparison to (8).

### Yield functions applied

#### Volumetric growth model for pine

As the first practical example, a recently introduced [10] yield function is considered, applicable to average pine stands in Northern Sweden. A volumetric growth function is

$$V\left(a\right)=580.14*{\left(1-6.3582^{-a/95}\right)}^{2.8967}.$$ (11)

The application introduced by Gong and Löfgren [10] assumes a volumetric stumpage price of 250 SEK/m^3^ and an initial investment of 6000 SEK/ha. The maximum sustainable volumetric yield rotation is 95 years, corresponding to that duration of time that gives the greatest average annual growth [10]. A 3% discount interest applied in Eq (9) yields an optimal rotation age of 52 years [10].

#### Value growth model for pine

Eq (11) does not consider any variation of the volumetric stumpage price. In other words, the value growth corresponds to volumetric growth, multiplied by a constant. That may be an unrealistic assumption, for a variety of reasons, including harvesting expenses as well as industrial use of the crop. In order to release this assumption, Gong and Löfgren [10] established an age-dependent price function

$$p\left(a\right)=104.63*{\left(a-29\right)}^{0.2602}.$$ (12)

We here apply Eq (12), for *a*>29, in addition to Eq (11), in order to establish another version of the practical forestry example. Maximum sustainable value yield is gained at 130 years of rotation. A 3% discount interest applied in Eq (9) yields an optimal rotation age of 62 years [10].

#### Value growth model for spruce

The third empirical model describes fertile spruce forests with natural regeneration. The growth model does not refer to the age of trees; basal area and tree diameter distribution are essential independent variables [35, 36]. Correspondingly, the stand age does not refer to the age of trees, but to the time elapsed after the latest regeneration harvesting within any stand. The empirical model of Bollandsås et al. [35, 36] is applied and parametrized with reference to a recently introduced natural stationary state [41, 40].

Any regeneration harvesting is defined with respect to a natural stationary state [41, 40]. In particular, a diameter-limit cutting to breast height diameter 25 cm is introduced, the number of remaining trees within any diameter class below 25 cm adjusted to the number of trees in the natural stationary state [41, 40]. Then, any stand is allowed to develop according to the empirical model by Bollandsås *et al*. [35, 36], until to a rotation age, where another regeneration harvesting is assumed to occur. Time is discussed in terms of five-year increments. The model is parametrized with site fertility index 20, which corresponds to the dominant height in meters for an even-aged stand of 40 years of age [35, 36].

**Fig 1:**
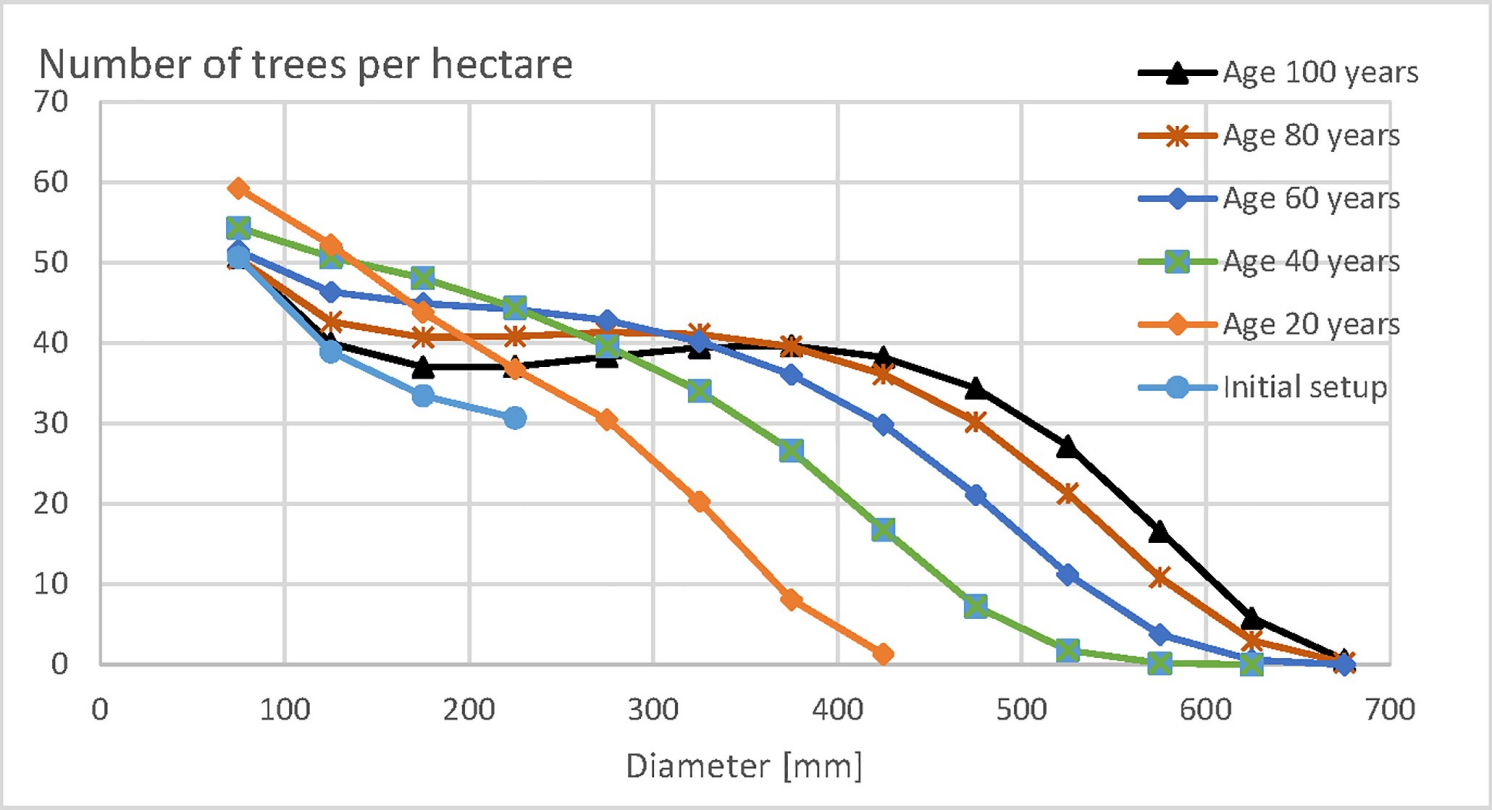
Number of trees in 50 mm diameter classes in the initial setup, as well as later stages of stand development, according to the spruce growth model [35, 36, 41, 40]. Stand age refers to the time elapsed after the latest regeneration harvesting.

The number of trees per hectare in different diameter classes, according to the model described above, is shown in Fig 1. The initial setup naturally contains only trees of diameter less than 250 mm. The number of trees in any diameter class, except the smallest, is smaller than in developed stages. A consequence is that the regeneration harvesting not only contains diameter-limit cutting, but also some thinning of smaller trees.

On the basis of the number of trees per hectare, the volumetric amount of two assortments, pulpwood and sawlogs, is clarified according to an appendix given by of Rämö and Tahvonen [42, 43]. The monetary value of the assortment volumes is clarified according to stumpage prices given by Rämö and Tahvonen [42]. In the initial setup, the number of trees (breast-height diameter at least 50 mm) per hectare is 154. The basal area is 2.72 m^2^/ha, and the combined volume of the assortments 18.5 m^3^/ha. The stumpage value of the trees in the initial setup is 670 Eur/ha.

### Analytical procedures

For any of the three growth models (Eq (11), and (11) combined with (12), and [35, 36, 41, 40], the rotation age is optimized according to any of the three different objective functions given in Eqs (9), (10) and (8). In order to keep the treatment simple, no intermediate revenues or expenses within a rotation are discussed.

Maximization of the net present value of proceeds according to Eq (9) requires an external discount interest rate. Three different rates are adopted, 2%, 3%, and 4%. Maximization of the internal rate of return according to Eq (10), as well as the expected value of capital return according to Eq (8) require a bare land value, as an opportunity cost in Eq (10) and as a part of the applied capitalization in Eq (8). Three different bare land values are applied: zero, a moderate, and a high bare land value. In the case of pine stand models with a regeneration expense, the moderate bare land value equals half of the regeneration expense, and the high value two times the regeneration expense. In the case of the spruce model with natural regeneration, the moderate bare land value is 70% of the value of trees in the initial setup, and the high value 300% of the value of trees in the initial setup. Since stationary forestry is discussed, any appreciation of the real estate prices is not considered.

After determining the optimal rotation age for three different growth models, using the three objective functions, we find out what kind of expected value of relative wealth increment rate those rotation ages yield according to Eq (8). Again, this is done using the three different bare land values as explained above. Eq (8) of course can be substituted to Eq (7) or to Eq (2).

In order to apply Eq (8), an amortizations schedule for any eventual initial investment is needed. We choose to make a one-time amortization at the end of any rotation, simultaneously as the final proceeds are gained. A consequence is that there is no amortization of initial investment affecting the denominator of Eq (8); the investment expense contributes to the capitalization during the entire rotation. On the other hand, the change of capitalization during the rotation, appearing in the numerator of Eq (8), is net of regeneration expense.

## Results

### Pine volumetric growth model

**Fig 2:**
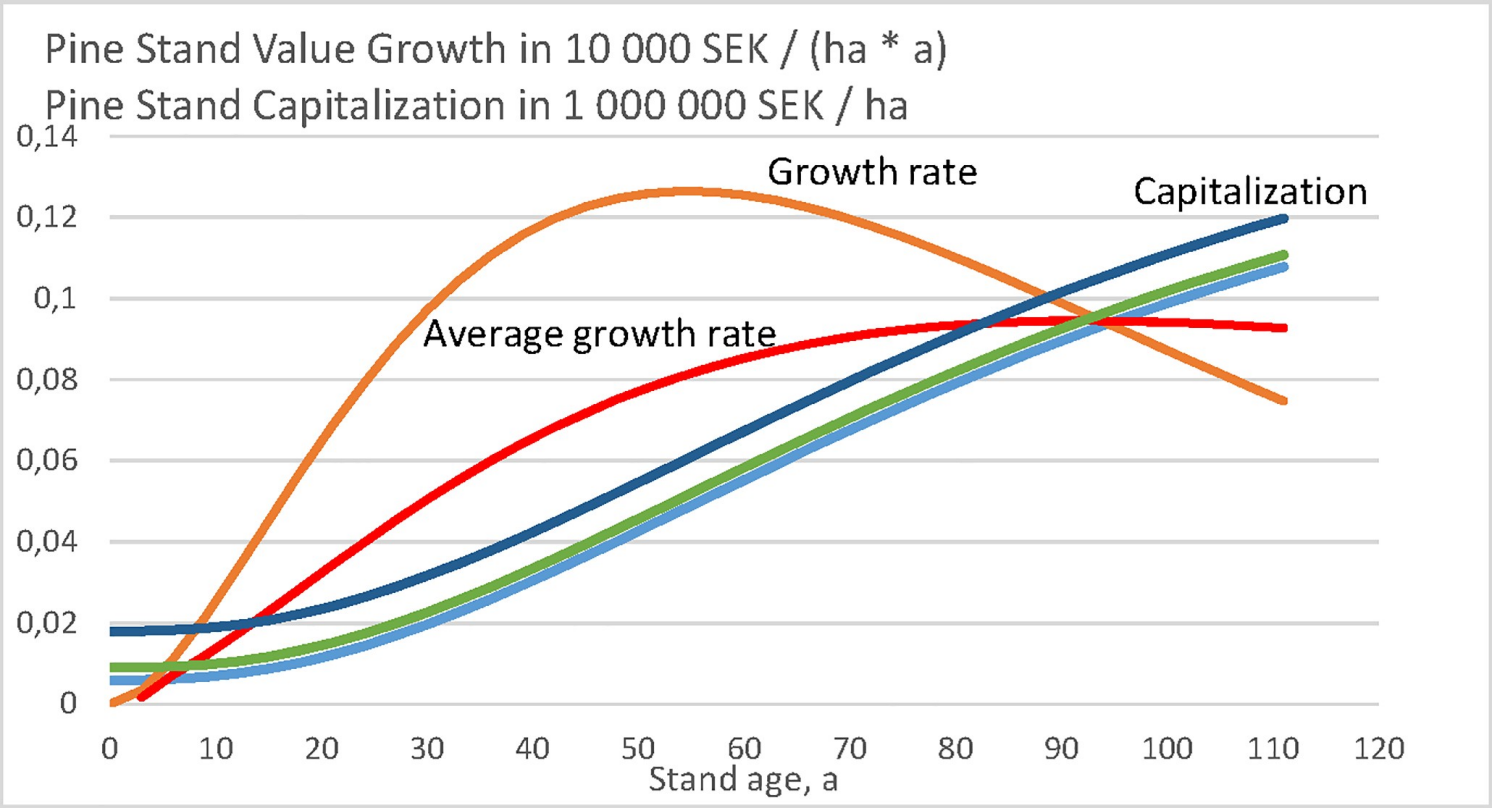
Pine stand value growth as a function of stand age, according to a North-Swedish growth function (11), [10]. Value growth is given in units of 10 000 SEK, whereas the capitalization in millions per hectare. Three curves of capitalization correspond to bare land values 0, 3000 SEK/ha and 12 000 SEK/ha.

Let us first plot the momentary capitalization *κ*(*a*), as well as the increment rate of momentary capitalization $\frac{d\kappa }{da}$ as a function of stand age *a* in Fig 2. The result is an outcome of the growth model only and does not depend on the objective function applied. The increment rate of capitalization does not depend on the bare land value, whereas the capitalization itself does. It is found that the average increment rate of capitalization reaches the momentary increment rate at stand age 95 years, which corresponds to the rotation age of maximum sustainable volumetric yield.

**Fig 3:**
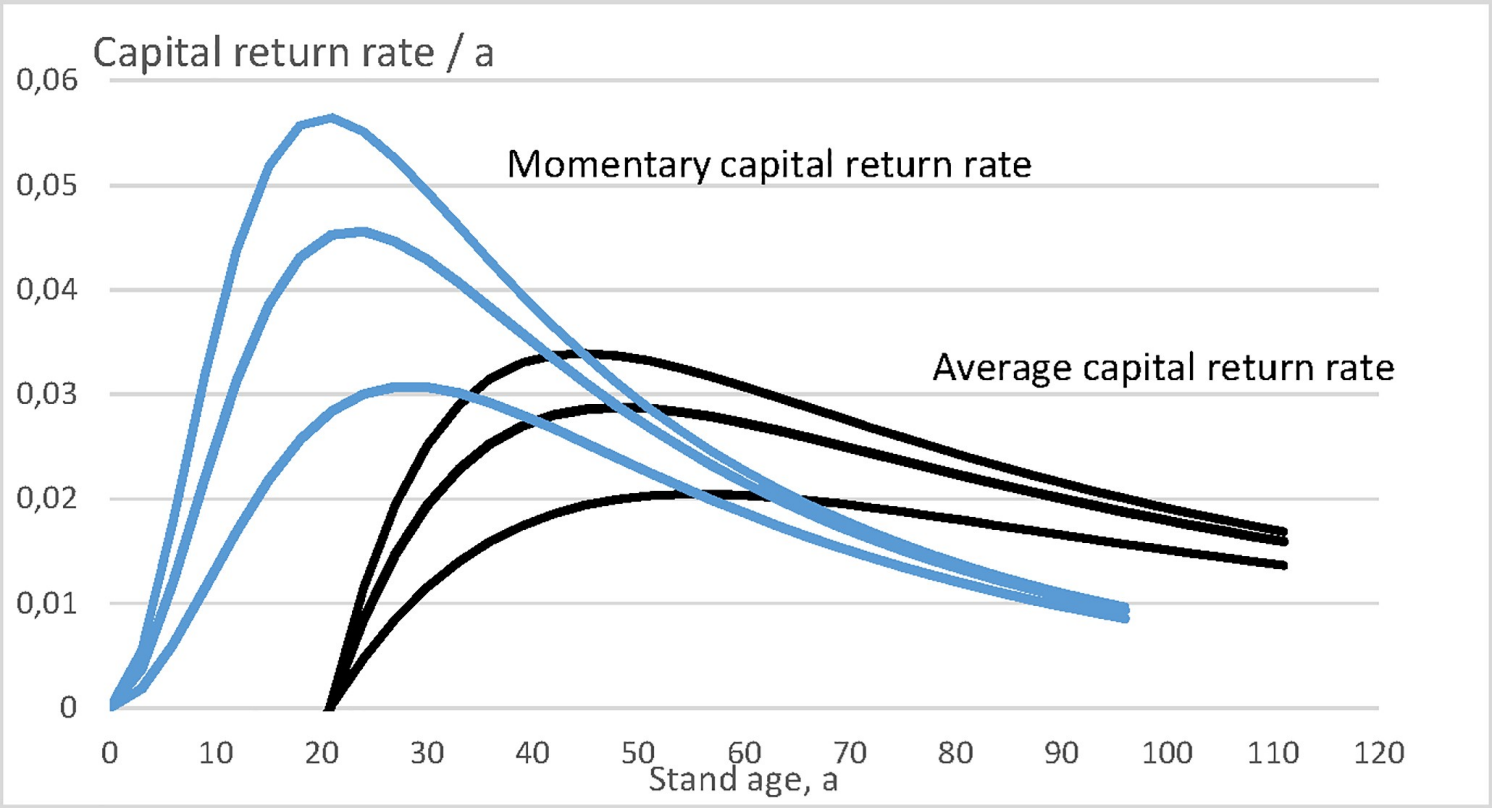
Momentary capital return rate $r(a)=\frac{d\kappa (a)}{\kappa (a)dt}$, as well as expected wealth appreciation rate 〈*r*〉 (Eq (8)), for three bare land values 0, 3000 SEK/ha and 12 000 SEK/ha.

Let us then plot the momentary capital return rate $r(a)=\frac{d\kappa (a)}{\kappa (a)dt}$ as a function of stand age *a*, and the expected value of the wealth appreciation rate 〈*r*〉 (Eq (8)) as a function of rotation age *τ*. These both are drawn in Fig 3. It is found that for any of the three bare land values, the expected value of the wealth appreciation rate reaches the momentary value at the maximum value of the former. The regeneration expense, as well as the bare land value taken as constants, the actual wealth appreciation rate depends on the rotation age according to Eq (8). The optimal rotation ages for bare land values 0, 3000 SEK/ha and 12 000 SEK/ha are 45, 48 and 56 years, corresponding to wealth appreciation rates to 3.396%, 2.877% and 2.047% per annum.

It is worth noting that in Fig 3, the momentary capital return rate is given as a function of stand age *a*, without any reference to any rotation age *τ*. We chose to make a one-time amortization at the end of any rotation, and the momentary capital return rate does not contain any amortization. On the other hand, the wealth appreciation rate 〈*r*〉 is given as a function of rotation age, and correspondingly the amortization contributes to it. That is why 〈*r*〉 first becomes nonnegative at rotation age of 21 years.

Let us then consider the objective functions (9) and (10). According to Eq (9), the optimal rotation ages are 60, 52 and 45 years with discounting interest rates 2%, 3%, and 4%, respectively. The corresponding net present values are 12 700, 2 830 and -1 150 SEK/ha. The negativity of the net present values would disqualify the largest discounting interest rate in most applications. The moderate bare land value of SEK 3000 is gained with 2,97% discounting interest, at rotation age 52 years. The high bare land value of SEK 12 000 is gained with 2.043% discounting interest, at rotation age 60 years. Zero bare land value is gained with 3.624% discounting interest, at rotation age 48 years.

According to Eq 10, the internal rate of return reaches its maximum value at rotation age 52 with the moderate bare land value and 60 with the high bare land value. The corresponding internal return rates are 2.972% and 2.043%. In the absence of any bare land value, the internal rate of return would be 3.624% at rotation age 48 years.

Obviously, the three different objective functions (Eqs (8), (9) and (10)) give different optimal rotation ages. With zero bare land value, the rotation ages are 45, 48 and 48 years. The corresponding annual wealth accumulation rates, computed from Eq (8), are 3.396%, 3.373% and 3.373%. These returns possibly should be contrasted to the outcome of Eq (9) with 2% and 3% discounting interests, which would give optimal rotation ages 60 and 52 years. These rotation ages, according to Eq (8), give capital appreciation rates 3.075% and 3.300%, respectively.

With the moderate bare land value of 3000 SEK/ha, the rotation ages are 48, 52 and 52 years. The corresponding annual wealth accumulation rates, computed from Eq (8), are 2.877%, 2.857% and 2.857%. These returns possibly should be contrasted to the outcome of Eq (9) with 2% and 3% discounting interests, which again give optimal rotation ages 60 and 52 years. These rotation ages, according to Eq (8) but now with the bare land value 3000 SEK/ha, give capital appreciation rates 2.726% and 2.857%, respectively.

With the high bare land value of 12 000 SEK/ha, the rotation ages are 56, 60 and 60 years. The corresponding annual wealth accumulation rates, computed from Eq (8), are 2.047%, 2.035% and 2.035%. These returns again should be contrasted to the outcome of Eq (9) with 2% and 3% discounting interests, which again give optimal rotation ages 60 and 52 years. These rotation ages, according to Eq (8) but now with the bare land value 12 000 SEK/ha, give capital appreciation rates 2.035% and 2.037%, respectively.

With this growth model, Eq (10) gives rotation ages and wealth appreciation rates rather close to the optimum. Since the outcome of Eq (9) depends on the discounting interest rate, the results vary. In the case of zero and moderate bare land values, the outcome with 2% discounting interest differs from the optimal. In the of the high bare land value, the outcome of Eq (9) differs only slightly from the optimum, with any of the two discounting interest rates.

Rather interestingly, the results from Eqs (9) and (10), regarding rotation time and wealth appreciation rate, are identical provided the discounting interest rate in Eq (9) is adjusted to result in any known bare land value as a net present value of future proceeds. The adjusted discounting interest rate in Eq (9) is the same as the internal rate of return in Eq (10). This leads us to suspect that the two objective functions possibly are identical.

### Pine value growth model

**Fig 4:**
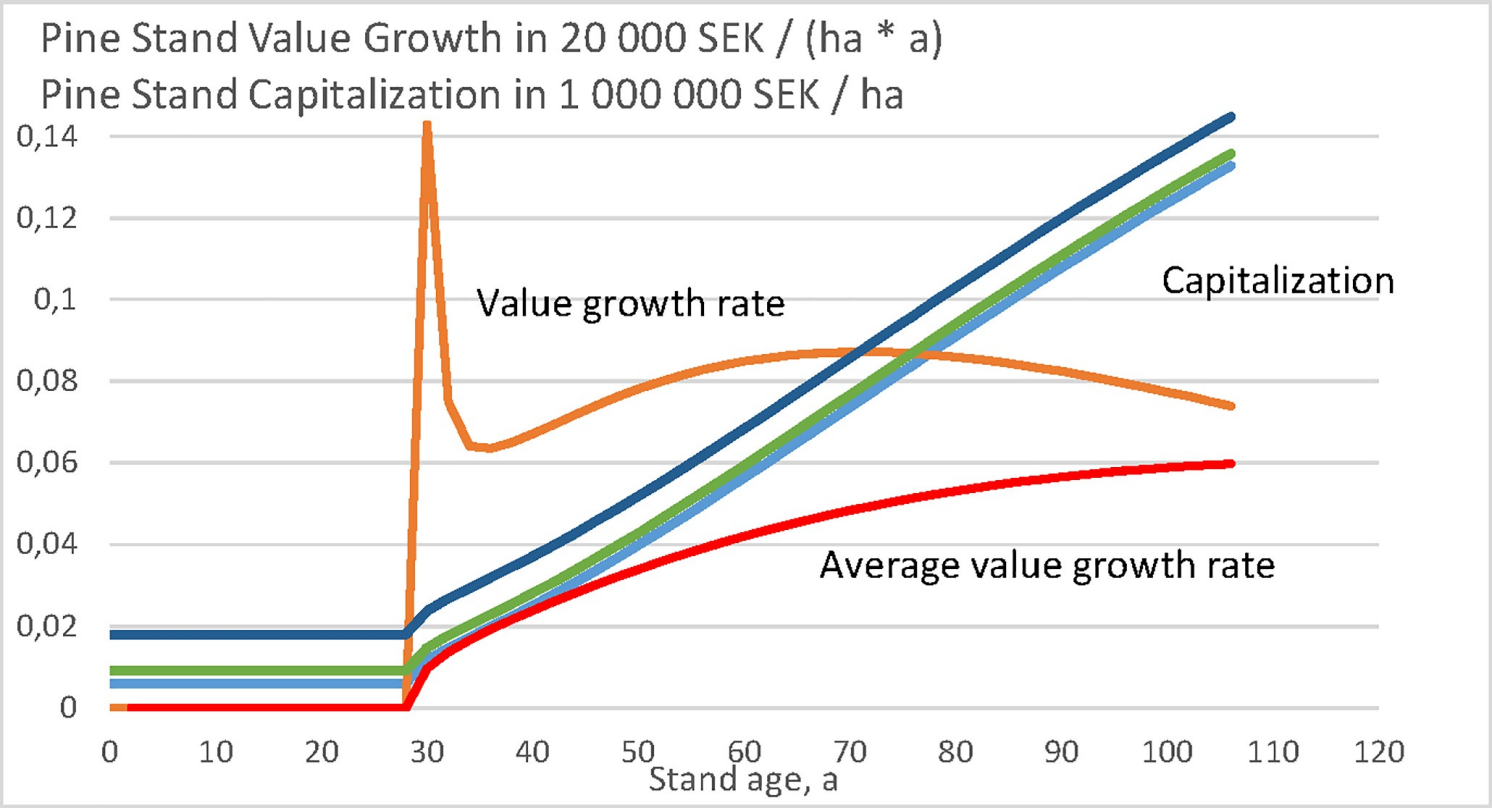
Pine stand value growth as a function of stand age, according to North-Swedish growth functions (11) and (12), [10]. Value growth is given in units of 20 000 SEK, whereas the capitalization in millions per hectare. Three curves of capitalization correspond to bare land values 0, 3000 SEK/ha and 12 000 SEK/ha.

Let us plot the momentary capitalization *κ*(*a*), as well as the increment rate of momentary capitalization $\frac{d\kappa }{da}$ for the value growth model given in Eqs (11) and (12), as a function of stand age *a* in Fig 4. Again, the result is an outcome of the growth model only and does not depend on the objective function applied. The increment rate of capitalization does not depend on the bare land value, whereas the capitalization itself does. The capitalization increases rapidly at the age of 30 years because of the step function (12). The average increment rate of capitalization reaches the momentary increment rate at stand age 130 years, which corresponds to the rotation age of maximum sustainable value yield.

**Fig 5:**
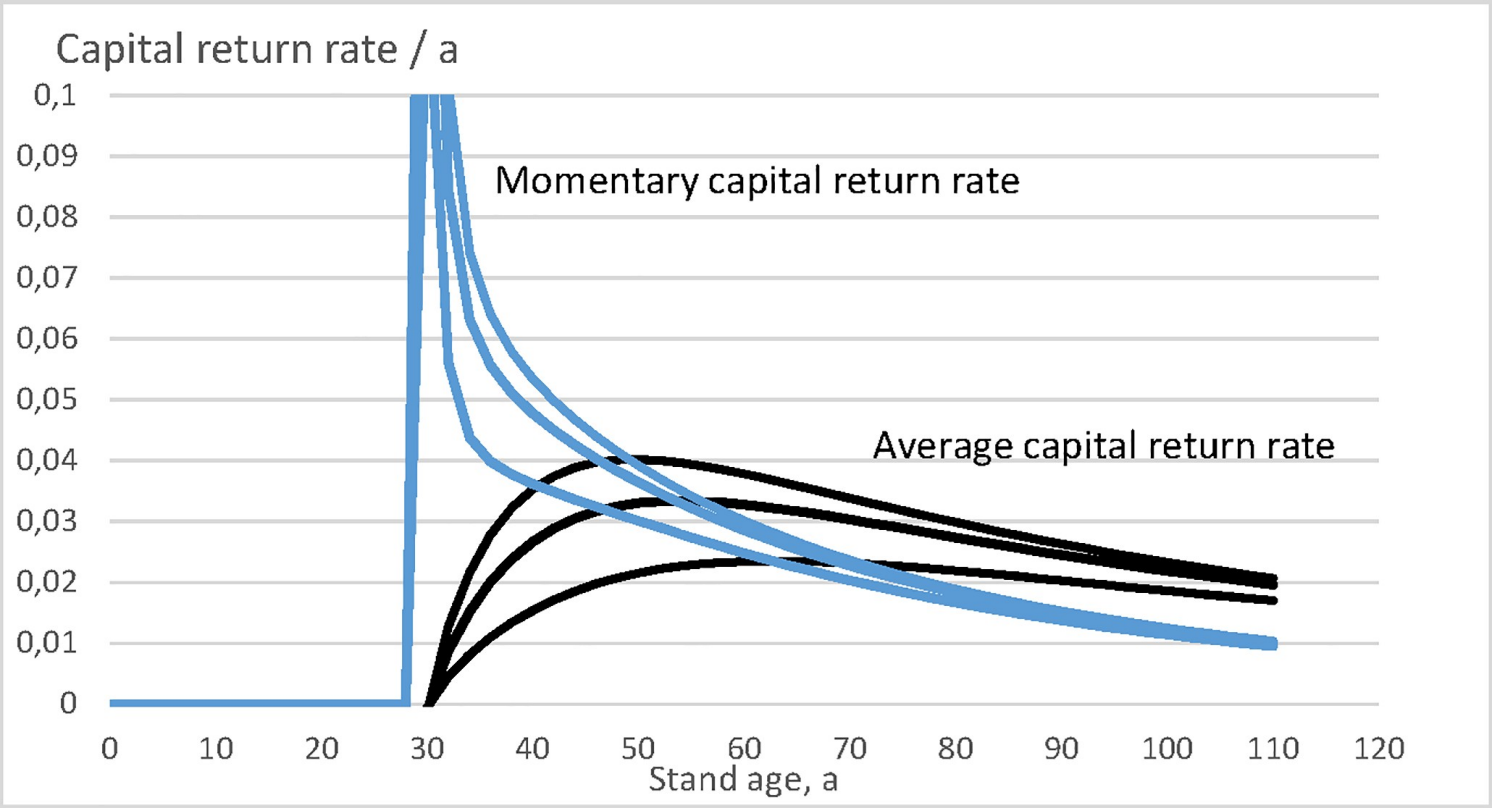
Momentary capital return rate $r(a)=\frac{d\kappa (a)}{\kappa (a)dt}$, as well as expected wealth appreciation rate 〈*r*〉 (Eq (8)), for three bare land values 0, 3000 SEK/ha and 12 000 SEK/ha.

Let us then plot the momentary capital return rate $r(a)=\frac{d\kappa (a)}{\kappa (a)dt}$ as a function of stand age *a*, and the expected value of the wealth appreciation rate 〈*r*〉 (Eq (8)) as a function of rotation age *τ*. These both are drawn in Fig 5. It is again found that for any of the three bare land values, the expected value of the wealth appreciation rate reaches the momentary value at the maximum value of the former. The regeneration expense, as well as the bare land value taken as constant, the actual wealth appreciation rate depends on the rotation age according to Eq (8). The optimal rotation ages for bare land values 0, 3000 SEK/ha and 12 000 SEK/ha are 49, 54 and 63 years, corresponding to wealth appreciation rates to 4.034%, 3.347% and 2.354% per annum.

It is worth noting that in Fig 5, the momentary capital return rate is given as a function of stand age *a*, without any reference to rotation age *τ*. We chose to make a one-time amortization at the end of any rotation, so the momentary capital return rate does not contain any amortization. On the other hand, the wealth appreciation rate 〈*r*〉 is given as a function of rotation age, and correspondingly the amortization contributes to it. That is why 〈*r*〉 first becomes nonnegative at rotation age of 31 years.

Let us then consider the objective functions (9) and (10). According to Eq (9), the optimal rotation ages are 73, 62 and 56 years with discounting interest rates 2%, 3%, and 4%, respectively. The corresponding net present values are 14 200, 2 820 and -1 120 SEK/ha. The negativity of the net present values would disqualify the largest discounting interest rate in most applications. The moderate bare land value of SEK 3000 is gained with 2.972% discounting interest, at rotation age 63 years. The high bare land value of SEK 12 000 is gained with 2.126% discounting interest, at rotation age 71 years. Zero bare land value is gained with 3.549% discounting interest, at rotation age 58 years.

According to Eq (10), the internal rate of return reaches its maximum value at rotation age 63 with the moderate bare land value and 71 with the high bare land value. The corresponding internal return rates are 2.972% and 2.126%, respectively. In the absence of any bare land value, the internal rate of return would be 3.549% at rotation age 58 years.

Obviously, the three different objective functions (Eqs (8), (9) and (10)) shall give different optimal rotation ages. With zero bare land value, the rotation ages are 49, 58 and 58 years. The corresponding annual wealth accumulation rates, computed from Eq (8), are 4.034%, 3.859% and 3.859%. With the moderate bare land value of 3000 SEK/ha, the rotation ages are 54, 63 and 63 years. The corresponding annual wealth accumulation rates, computed from Eq (8), are 3.347%, 3.222% and 3.222%. With the high bare land value of 12 000 SEK/ha, the rotation ages are 63, 71 and 71 years. The corresponding annual wealth accumulation rates, computed from Eq (8), are 2.354%, 2.308% and 2.308%.

Interestingly, the results based on Eqs (9) and (10) are again identical. This is the case provided the discounting interest used in Eq (9) is calibrated to gain the same bare land value as is used as the input variable in Eq (10). The situation is different if arbitrary discounting interests are used in Eq (9). With 2% and 3% discounting interests, the optimal rotation ages would be 73 and 62 years, respectively. According to Eq (8), these rotation ages would yield capital appreciation rates 3.263% and 3.713% for zero bare land value, 2.948% and 3.244% for moderate bare land value, and 2.286% and 2.353% for the high bare land value. In the case of zero and moderate bare land values, the outcome with 2% discounting interest again differs from the optimal, this time more severely than in the case of the volumetric growth model discussed above.

### Spruce value growth model

**Fig 6:**
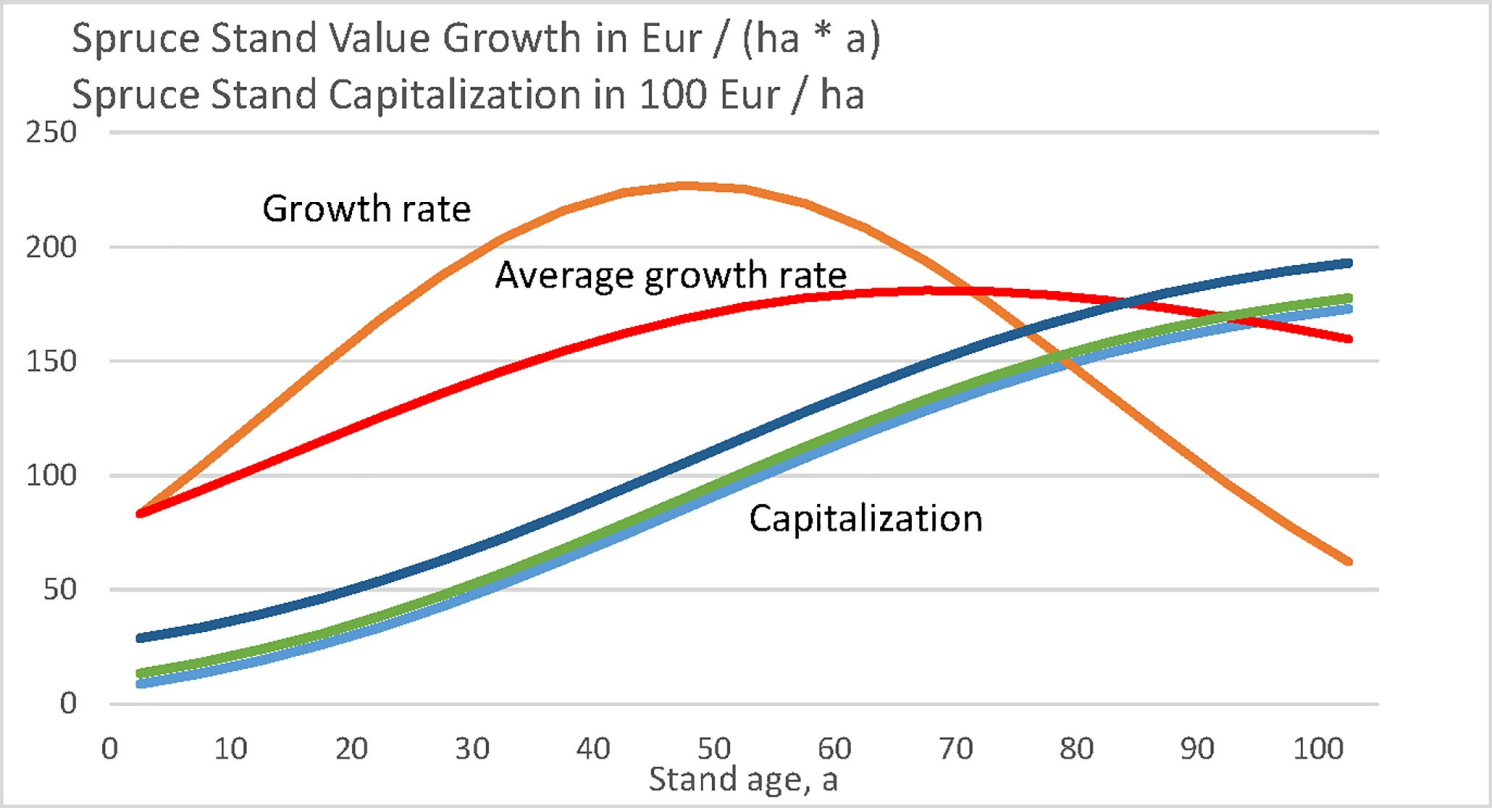
Spruce stand value growth as a function of stand age, according to Bollandsås et al. (Fig 1, [35, 36, 41, 40]). The capitalization is given in hundreds of Euros per hectare. Three curves of capitalization correspond to bare land values 0, 469 Eur/ha and 2009 Eur/ha.

Let us plot the momentary capitalization *κ*(*a*), as well as the increment rate of momentary capitalization $\frac{d\kappa }{da}$ for the spruce value growth model [35, 36, 41, 40], as a function of stand age *a* in Fig 6. Again, the result is an outcome of the growth model only and does not depend on the objective function applied. The increment rate of capitalization does not depend on the bare land value, whereas the capitalization itself does. The average increment rate of capitalization reaches the momentary increment rate at stand age 70 years, which corresponds to the rotation time of maximum sustainable value yield.

**Fig 7:**
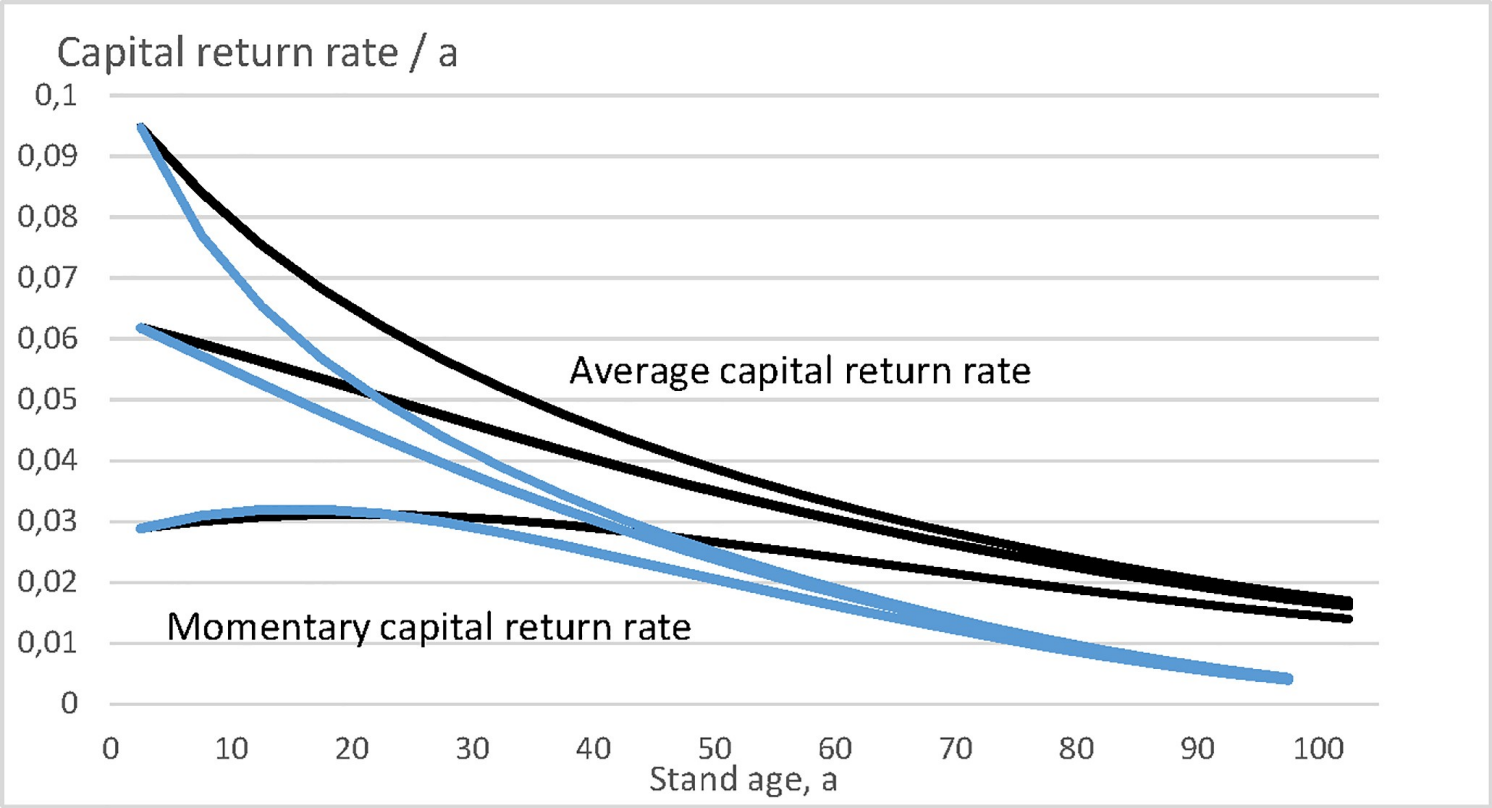
Momentary capital return rate $r(a)=\frac{d\kappa (a)}{\kappa (a)dt}$, as well as expected wealth appreciation rate 〈*r*〉 (Eq (8)), for three bare land values 0, 469 Eur/ha and 2 009 Eur/ha.

Let us then plot the momentary capital return rate $r(a)=\frac{d\kappa (a)}{\kappa (a)dt}$ as a function of stand age *a*, and the expected value of the wealth appreciation rate 〈*r*〉 (Eq (8)) as a function of rotation time *τ*. These both are drawn in Fig 7. With bare land values 0 and 469 Eur/ha the capital return rate reaches its maximum during the first five-year growth period, being 9.48% and 6.18%. With bare land value 2 009 Eur/ha the wealth appreciation rate reaches its maximum during the fifth five-year growth period, being 3.12%.

Let us then consider the objective functions (9) and (10). According to Eq (9), the optimal rotation times are 40, 25 and 10 years with discounting interest rates 2%, 3%, and 4%, respectively. The corresponding net present values of the initial setup (bare land and trees standing after the regeneration harvesting) are 5062, 2821 and 1905 Eur/ha. The moderate initial setup value of Eur/ha 1139 is gained with 6.24% discounting interest, at rotation time 5 years. The high initial setup value of Eur 2679/ha is gained with 3.11% discounting interest, at rotation time 25 years. Initial setup value consisting only of trees remaining after the regeneration cutting is gained with 9.68% discounting interest, at rotation time 5 years.

According to Eq (10), the internal rate of return reaches its maximum value at rotation time 5 with the moderate bare land value and 25 with the high bare land value. The corresponding internal return rates are 6.24% and 3.11%, respectively. In the absence of any bare land value, the internal rate of return would be 9.68% at rotation time 5 years.

Again, the results based on Eqs (9) and (10) are identical, provided the discounting interest in Eq (9) is calibrated to produce a known net present value for the initial setup. In the case of this spruce growth model, also direct optimization according to Eq (8) gives the same rotation times. Internal rates of return resulting from Eq (10) slightly differ from wealth accumulation rates coming from Eq (8), the former Equation neglecting the shape of the yield curve, whereas Eq (8) does not. The discounting interest rates needed in Eq (9) to produce known initial setup values as net present values are the same as the internal return rates in Eq (8).

The economic views diverge if arbitrary discounting interests are used in Eq (9). With 2%, 3% and 4% discounting interests, the optimal rotation times would be 40, 25 and 10 years, respectively. According to Eq (8), these rotation times would yield capital appreciation rates 4.76%, 6.21% and 8.41% for zero bare land value, 4.16%, 5.04% and 5.92% for moderate bare land value, and 2.94%, 3.12% and 3.00% for the high bare land value.

## Discussion

Two of the objective functions discussed yield financially satisfactory results, whereas the third is clearly inferior. However, the inferior method of maximization of net present value appear to yield numerical results identical to those of internal rate of return, provided that the discounting interest in Eq (9) is calibrated to yield an appropriate bare land value. This raises a question whether the two objective functions are identical with the mentioned boundary condition. Eq (10) can readily be rewritten for the special case where there are nonzero net proceeds a two time instants: at the beginning of each growth cycle, and at the end of the growth cycle. In the beginning, there is an initial investment *I* and a purchase expense for the bare land *B*. At the end of the cycle, there is harvesting revenue *R*, and sales proceeds for the bare land *B*. Then, Eq (10) can be rewritten as

$$\left(R+B\right)e^{-o\tau }-\left(I+B\right)=0.$$ (13)

Resolving the bare land value yields

$$B=\left(Re^{-o\tau }-I\right)\frac{1}{1-e^{-o\tau }}.$$ (14)

Eq (12) however is the same as Eq (9), with the same boundary conditions. One can readily show that the equality applies to any schedule of proceeds where a resource is occupied at the beginning of a period and released at the end. The similarity of Eqs (13) and (9) is particularly interesting since Eq (13) corresponds to one rotation, whereas Eq (9) contains a factor considering all growth cycles up to a distant future. We look forward for investigating this in more detail in a consequent paper.

Eqs (13) and (14) demonstrate that two of the above-discussed objective functions are the same, provided the discounting interest rate in Eq (9) is adjusted to yield an appropriate bare land value. Then, one must ask how can one clarify what is an appropriate bare land value? In the mind of the author, this is not too difficult. There is bare forest land available in the real estate market. There also are young plantations on the market, and the bare land value is achievable by deducting their regeneration expense. On the other hand, there hardly is any unambiguous way of determining a valid discounting interest rate, apart from the calibration to yield a valid bare land value. No single market interest rate can be selected for the discounting interest; at the time of writing, mortgage interest rates within the Eurozone vary 1% &2%, but an opportunity cost for neglected alternative investments easily becomes 6% …9%. The range is far too wide.

**Fig 8:**
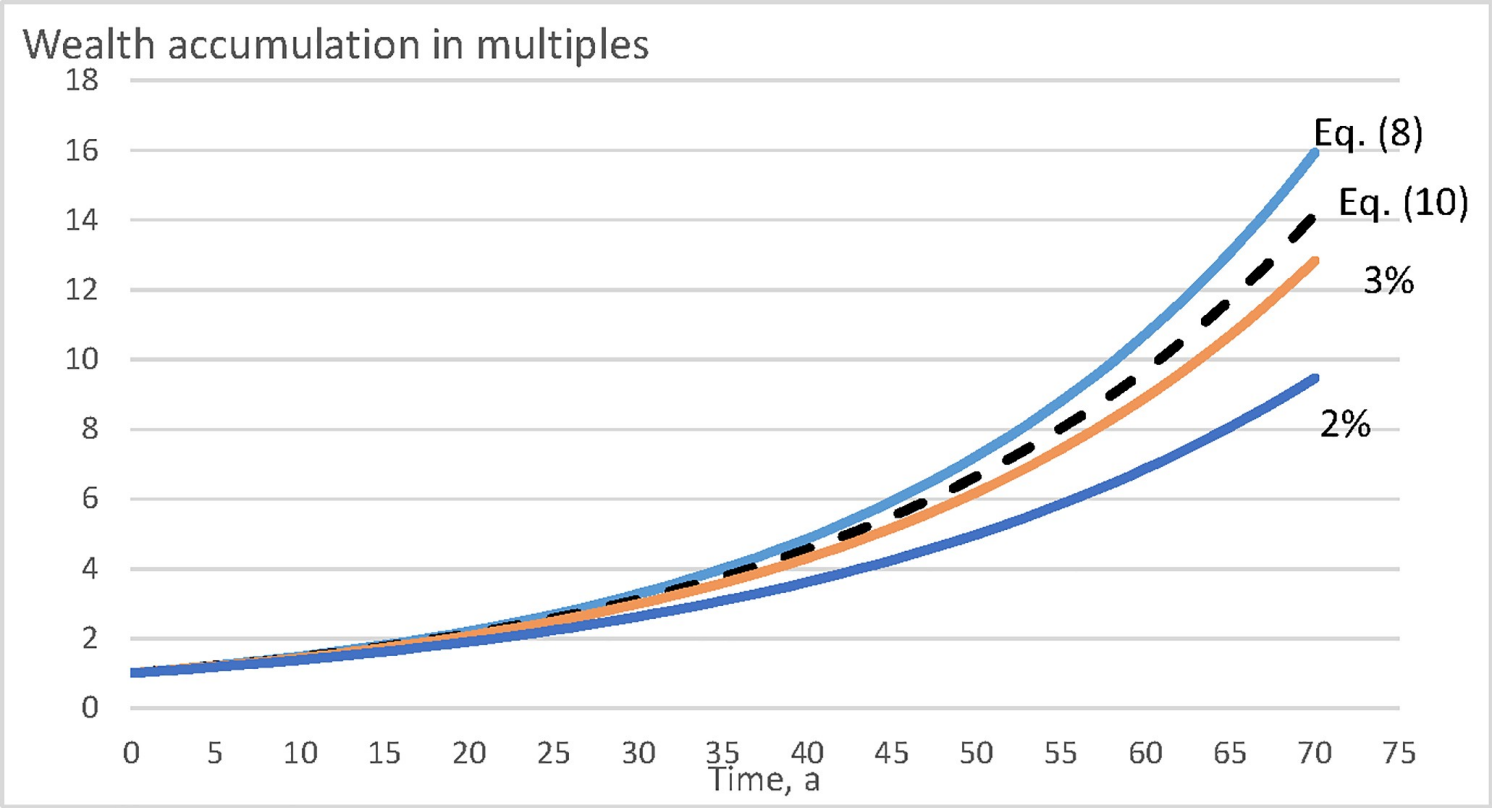
Wealth accumulation as a function of time (in years) for the value growth model introduced in Eqs (11) and (12), with bare land value 0 SEK/ha.

In a few cases, the wealth appreciation rate is not very sensitive to the objective function selected. However, there are rather sensitive cases. Fig 8 shows wealth accumulation within the value growth model described in Eqs (11) and (12), for a period of 70 years, in the absence of any bare land value. It is found that there are clearly observable differences between the different objective functions. Direct maximization of Eq (8) results as a rotation age of 49 years. Maximization of internal rate of return according to Eq (10) results as rotation age 58 years. Net present value maximization according to Eq (9) results as rotation ages 73 and 62 years, with 2% and 3% discounting interest, respectively. In Fig 8, wealth accumulation is only slightly suboptimal if rotation age is determined either with Eq (10) or with Eq (9) with discounting interest rate 3%. However, with discounting interest 2%, Eq (9) yields clearly inferior results.

**Fig 9:**
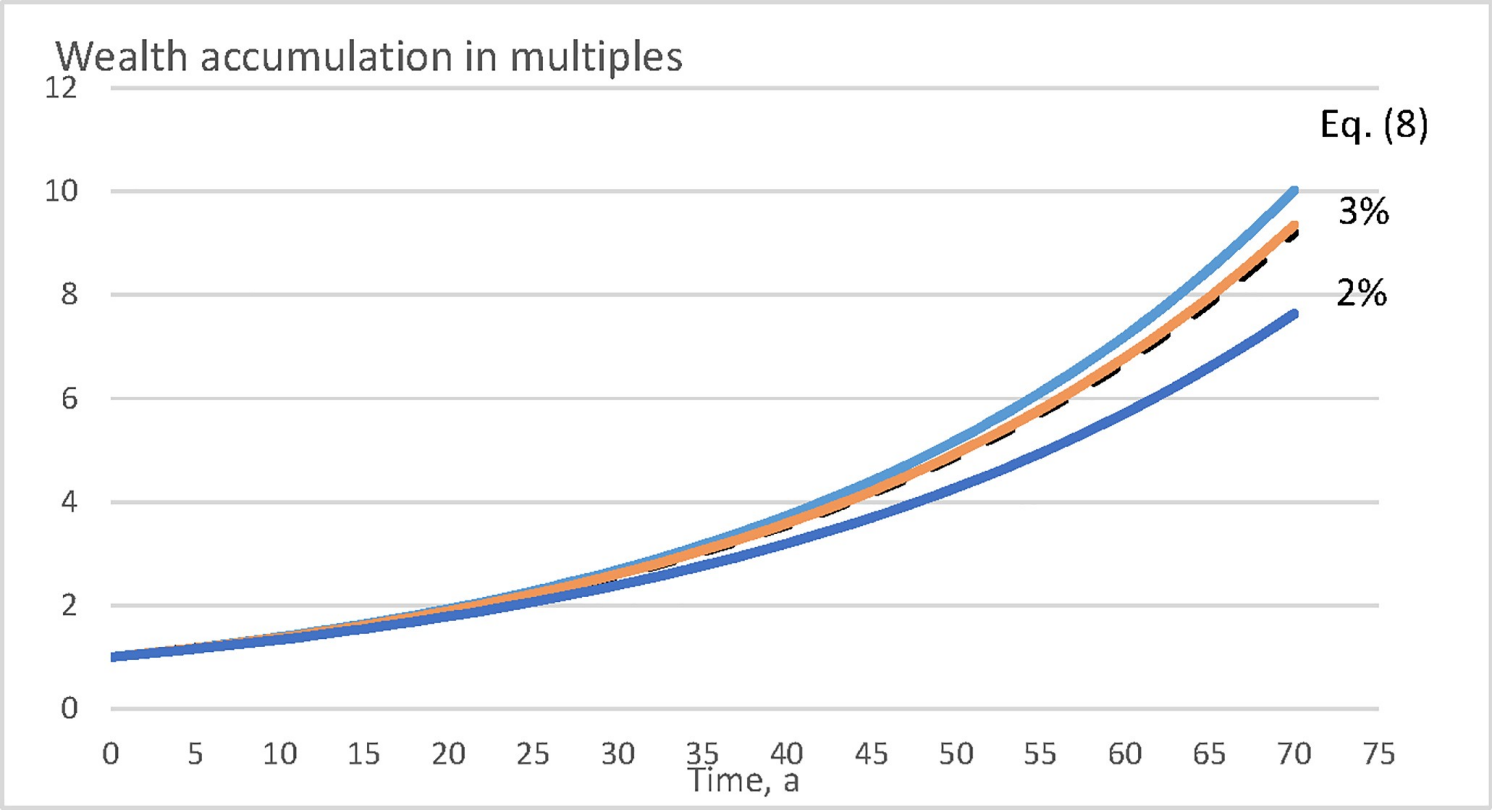
Wealth accumulation as a function of time (in years) for the value growth model introduced in Eqs (11) and (12), with moderate bare land value 3000 SEK/ha. The dotted line corresponds to the internal rate of return optimization according to Eq (10).

Fig 9 shows wealth accumulation within the value growth model described in Eqs (11) and (12), for a period of 70 years, for the moderate bare land value 3000 SEK/ha. It is found that there again are clearly observable differences between the different objective functions, even if somewhat less than in Fig 8. Direct maximization of Eq (8) results as a rotation age of 54 years. Maximization of internal rate of return according to Eq (10) results as rotation age 63 years. Net present value maximization according to Eq (9) results as rotation ages 73 and 62 years, with 2% and 3% discounting interest, respectively. It is found from Fig 9 that Eq (9) with 3% discounting interest yields a slightly greater wealth accumulation rate than Eq (10). This is naturally accidental. The 3% discounting rate in Eq (9) being selected arbitrarily, it happens to result in one year younger rotation age in the case of Fig 9. Again, Eq (9) with discounting interest 2%, yields clearly inferior results.

**Fig 10:**
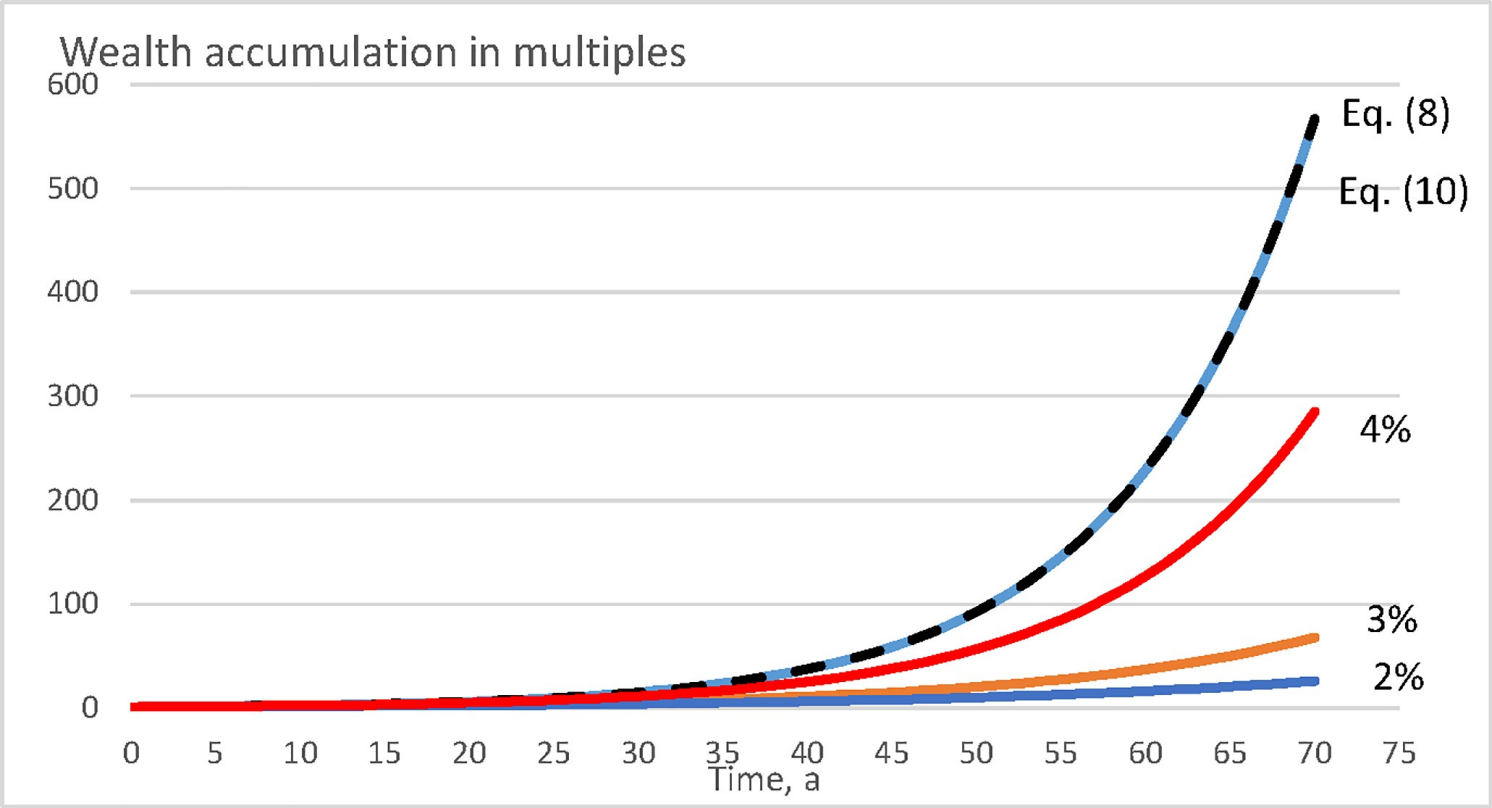
Wealth accumulation as a function of time (in years) for according to the spruce value growth model [35, 36, 41, 40], with bare land value 0 Eur/ha.

Fig 10 shows wealth accumulation within the spruce value growth model [35, 36, 41, 40], in the absence of any bare land value. It is found that there are large differences between the different objective functions. Direct maximization of Eq (8) results as a rotation time of five years. Maximization of internal rate of return according to Eq (10) results in the same rotation time. Net present value maximization according to Eq (9) results as rotation times 40, 25 and 10 years, with 2%, 3%, and 4% discounting interest, respectively. All these three discounting interest rates in Eq (9) result as financially inferior procedures. The lower the discounting interest is, the more devastating are the consequences.

**Fig 11:**
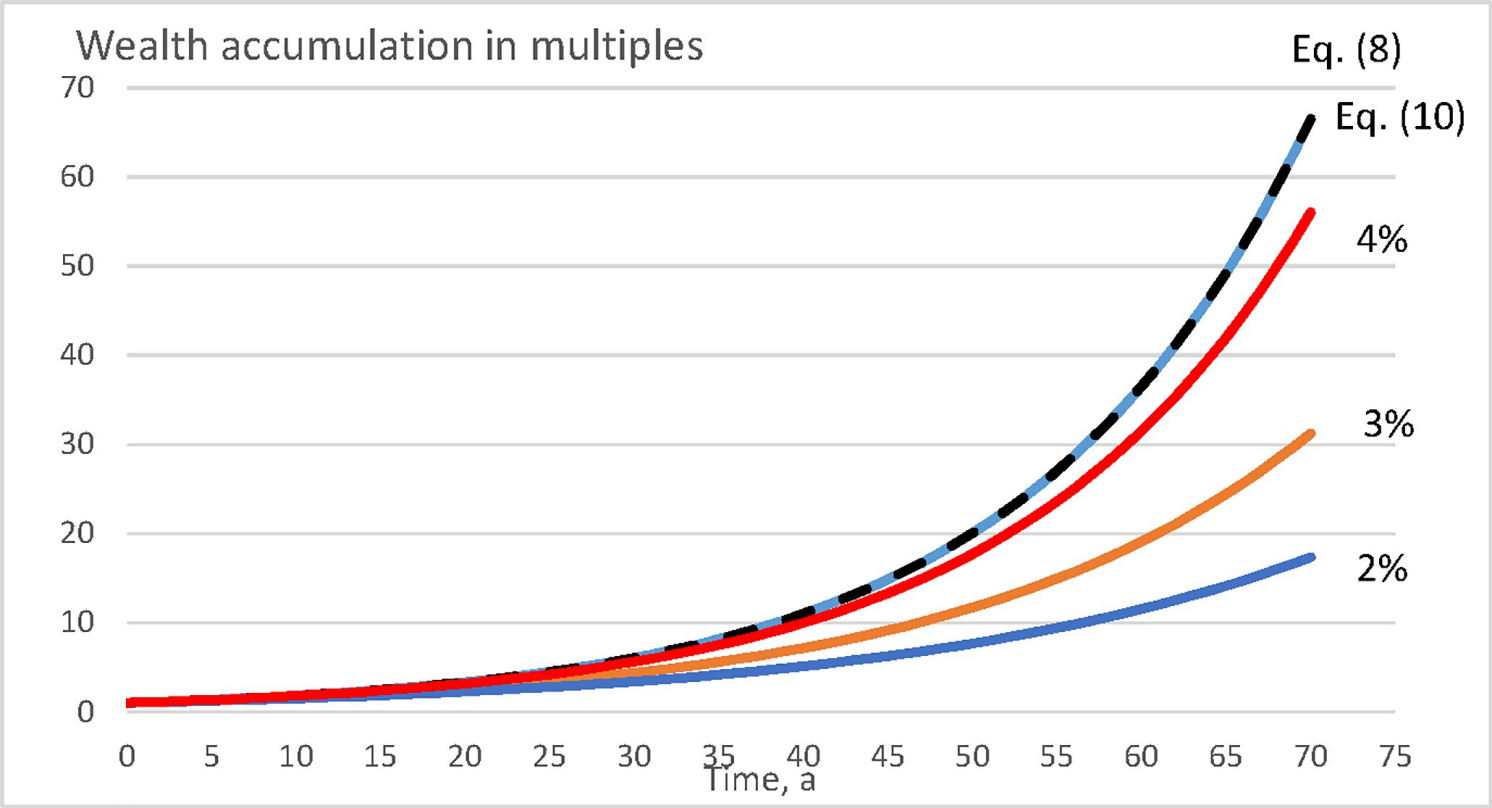
Wealth accumulation as a function of time (in years) according to the spruce value growth model [35, 36, 41, 40], with moderate bare land value 469 Eur/ha.

Fig 11 shows wealth accumulation within the spruce value growth model [35, 36, 41, 40], with the moderate any bare land value 469 Eur/ha. There are again large differences between the different objective functions. Direct maximization of Eq (8) results as a rotation time of five years. Maximization of internal rate of return according to Eq (10) results in the same rotation time. Net present value maximization according to Eq (9) results as rotation times 40, 25 and 10 years, with 2%, 3%, and 4% discounting interest, respectively. The lower the discounting interest is, the more devastating are the consequences. It is, however, worth noting that discounting interest rates exceeding the internal rate of return 6,23% would not produce any net present value matching the value of the initial setup.

It is of interest to consider why the maximization of the internal rate of return according to Eq (10) yields wealth appreciation rates lower than direct maximization of Eq (8). The reason naturally is that the IRR is determined on the basis of cash proceeds and neglects the details of the yield curve. The details of the yield curve, however, do contribute to the wealth accumulation rate. Regardless of this, maximizing the internal rate of return, in general, does not lead to any large deviation from financially optimal management procedures (Figs 8, 9, 10 and 11).

Unlike the internal rate of return, maximization of net present value with external discounting interest rate does induce large deviations from financially optimal management (Figs 8, 9, 10 and 11). However, consequences of the different discounting interest rates differ. There always is a discounting interest rate where maximizing the net present value becomes equal to maximizing the internal rate of return. However, arbitrarily adopting an external discounting interest leads to highly variable and at worst devastating financial consequences (Figs 8, 9, 10 and 11). This also applies to discounting interest rates commonly utilized in net present value computations in forestry [44, 42, 45, 46, 47, 48].

It is worth noting that a 3% discounting rate in Eq (9), relatively commonly used in forestry [45, 46, 47], performs relatively well in the pine growth models discussed above (Figs 8 and 9), but produces clearly inferior results in the case of the spruce value growth model (Figs 10 and 11). A natural explanation is that the capital return rate, as well as the internal rate of return, is greater in the spruce value growth case, and correspondingly they differ more from the 3% discounting interest. A possibly justified way of determining the discounting interest is to take it as an opportunity cost of an alternative investment, possibly from that giving the greatest internal rate of return within the business of forestry itself. As a consequence, Eqs (9) and (10) coincide, as shown in Eqs (13) and (14).

In this paper, stationary rotation forestry has been discussed. In other words, stand ages have been assumed to be evenly distributed. Prices and expenses have been treated as constants in real terms. It has been recently shown that an eventually appreciating real estate price possibly dictates financially sustainable management practices [40]. It would be of interest to investigate such an effect on the performance of the different objective functions.

The results of this paper have been applied since late 2018 by practitioners aware of the results at the time. Forestry practices have changed dramatically. The periodic temporal boundary condition, required by Eq (9), however seldom is met in practical operations. The actual implementation of the maximization of capital return rate has been designed accordingly [49].
